# Angle-Only Filtering of a Maneuvering Target in 3D

**DOI:** 10.3390/s22041422

**Published:** 2022-02-12

**Authors:** Mahendra Mallick, Xiaoqing Tian, Yun Zhu, Mark Morelande

**Affiliations:** 1 Independent Researcher, Anacortes, WA 98221, USA; 2 School of Automation Science and Engineering, Xi’an Jiaotong University, Xi’an 710049, China; tianxiaoqing2017@stu.xjtu.edu.cn; 3 School of Computer Science, Shaanxi Normal University, Xi’an 710062, China; yunzhu@snnu.edu.cn; 4 National Australia Bank, Melbourne, VIC 3000, Australia; m.morelande@gmail.com

**Keywords:** angle-only filtering in 3D, infrared search and track (IRST) sensor, maneuvering target tracking, cubature Kalman filter (CKF), Itô stochastic differential equation

## Abstract

We consider the state estimation of a maneuvering target in 3D using bearing and elevation measurements from a passive infrared search and track (IRST) sensor. Since the range is not observable, the sensor must perform a maneuver to observe the state of the target. The target moves with a nearly constant turn (NCT) in the XY-plane and nearly constant velocity (NCV) along the *Z*-axis. The natural choice for the NCT motion is to allow perturbations in speed and angular rate in the stochastic differential equation, as has been pointed out previously for a 2D scenario using range and bearing measurements. The NCT motion in the XY-plane cannot be discretized exactly, whereas the NCV motion along the *Z*-axis is discretized exactly. We discretize the continuous-time NCT model using the first and second-order Taylor approximations to obtain discrete-time NCT models, and we consider the polar velocity and Cartesian velocity-based states for the NCT model. The dynamic and measurement models are nonlinear in the target state. We use the cubature Kalman filter to estimate the target state. Accuracies of the first and second-order Taylor approximations are compared using the polar velocity-based and Cartesian velocity-based models using Monte Carlo simulations. Numerical results for realistic scenarios considered show that the second-order Taylor approximation provides the best accuracy using the polar velocity or Cartesian velocity-based models.

## 1. Introduction

Angle-only filtering in 2D and 3D finds many important applications in passive tracking [1,2,3,4,5,6,7,8,9,10,11,12,13]. The advantage of passive tracking over active tracking is that the presence of the passive sensor cannot be detected by the target. Passive tracking arises in submarine tracking using a passive sonar [1,11], passive ranging using an infrared search and track (IRST) sensor [2,4,5,8,12], passive radar tracking [4], satellite-to-satellite passive tracking [14], video tracking [15], etc. In this paper, we focus on tracking an aircraft using an IRST sensor on another aircraft. This problem is more difficult than the case where multiple sensors are used. The bearings-only filtering (BOF) problem in 2D has been extensively studied, and a vast number of publications exist in the research literature [1,10,16,17,18,19], Chapter 6 of [11,20]. However, research in the angle-only filtering (AOF) problem in 3D is limited compared to that in the bearings-only filtering problem.

Observability is a major issue for the BOF problem in 2D [21] and AOF problem in 3D. In the 2D problem, a four or five-dimensional state is estimated from bearings-only measurements for a non-maneuvering and maneuvering target, respectively. To observe the state of the target, the sensor must perform maneuvers with a motion of higher order than that of the target [18]. If a four-dimensional Cartesian state is used for the BOF problem in 2D for a non-maneuvering target, it has been observed that the extended Kalman filter (EKF) [22,23] diverges. Modified polar coordinates (MPC) [1,24,25] were formulated to overcome the divergence of the EKF. The components of the MPC are bearing, bearing-rate, range-rate divided by range, and the inverse of range [1]. The first three components of MPC are observable even before an ownship maneuver. MPC decouple the observable and unobservable components of the state vector and provide improved performance of the EKF. Use of MPC makes the dynamic model for the nearly constant velocity (NCV) model nonlinear and complex, but the measurement model becomes linear. In addition to the EKF, the unscented Kalman filter (UKF) [26], cubature Kalman filter (CKF) [27], Gaussian sum filter (GSF) [28], particle filter (PF) [11], uncorrelated conversion based filter (UCF) [20], etc. have also been applied to the BOF problem in 2D. In order to address the observability problem, the multiple model-based range-parametrized (RP) EKF (RP-EKF) was proposed by Peach [19] and Kronhamn [16]. In addition to the EKF, other filters can also be used in the RP framework.

Most of the existing work on the angle-only filtering problem in 3D is for a non-maneuvering target using the NCV model. The sensor must perform a maneuver to observe the target state. For a non-maneuvering target, the EKF using the Cartesian state for the AOF problem in 3D does not diverge [8], even though the filter diverges for the corresponding BOF in 2D [11]. In analogy with the MPC in 2D, Stallard proposed the modified spherical coordinates (MSC) in 3D [12,13]. The components of the MSC are elevation, elevation-rate, bearing, bearing-rate times cosine of elevation, the inverse of range, and range-rate divided by range. As in the case of MPC, the dynamic model for the NCV motion using MSC is nonlinear and complex. However, the measurement model is linear since bearing and elevation are components of MSC. The log spherical coordinates (LSC) [29] can also be used as an alternate to the MSC. The first five components of the LSC are the same as those of the MSC, but the inverse of range (sixth component) is replaced by the logarithm of the range. Many studies have shown that the EKF using the MSC (EKF-MSC) provides a better state estimation accuracy than the Cartesian EKF (CEKF) for the NCV motion [2,12,13].

Starting with the work of Stallard, the EKF-MSC was used in [2,12,13,30]. Karlsson and Gustafsson used the PF and compared the PF-based algorithms with the multiple model-based range-parametrized EKF (RP-EKF) using the Cartesian state and MSC in a number of tracking scenarios [6,7]. Their results showed the superiority of the PF-based algorithms over the RP-EKF-based algorithms.

In our previous work [29], we compared the EKF-MSC and EKF-LSC using the continuous-discrete filtering approach with the discrete-time CEKF. The results of this study show that the EKF-MSC and EKF-LSC have comparable accuracy and perform better than the discrete-time CEKF for low measurement accuracy. For high measurement accuracy, the discrete-time CEKF has higher state estimation accuracy than the EKF-MSC and EKF-LSC. Prior to our work in [8,31,32], the process noise using the MSC was modeled approximately. We proposed new algorithms using the MSC to model the process noise exactly. The AOF for the NCV motion can be solved in the following three possible ways [8,31,32]:

- Use the discrete-time NCV model with the Cartesian state vector (with linear dynamic model) and nonlinear measurement model;
- Use the exact discrete-time NCV model with the MSC (with nonlinear dynamic model) and linear measurement model;
- Use the MSC with approximate discretization of the continuous-time dynamic model (with nonlinear dynamic model) and linear measurement model.

In [8], we performed a comprehensive study of the AOF problem for a non-maneuvering target in 3D using the EKF, UKF, and PF with Cartesian state vector and MSC. In this study, new algorithms using the EKF, UKF, and PF with the MSC were formulated, and improved filter initialization algorithms for the Cartesian state and MSC were presented. Four versions of the PF were used in this work: the Cartesian bootstrap filter (CBF), bootstrap filter using MSC with exact dynamic model (BF-MSC(E)), bootstrap filter using MSC with an approximate dynamic model (BF-MSC(A)), and the optimal importance density-based PF using MSC with an approximate dynamic model (ODIPF-MSC(A)). The initial range between the target and the sensor in the scenarios in [8] is higher than that in [6,7]. Numerical results from this study indicate that the state estimation accuracy of the PF-based algorithms is inferior compared with that of the EKF and UKF-based algorithms. For the BOF problem in 2D Chapter 6 of [11], the measurement SD is of the order of a degree and the measurement time interval is about 30–60 s. In this scenario, a PF has one of the best state estimation accuracies Chapter 6 of [11,20]. Secondly, compared to the EKF, the PF-based algorithms have about two orders of magnitude higher computational cost. Thirdly, It is now well established that, when the measurement accuracy and data rate are high (which is true for the current problem), PFs do not offer any advantage over the EKF, UKF, and CKF [33,34,35]. Therefore, we did not consider the PF in this study.

A novel batch Bayesian weighted instrumental variable estimator for the 3D target motion analysis problem using bearing and elevation measurements is presented in [36]. Results of this study show that the proposed algorithm outperforms its non-Bayesian counterpart. The CEKF, Cartesian UKF (CUKF), Cartesian CKF (CCKF), and the Cartesian new sigma point Kalman filter (CNSKF) were used in [3] to analyze the AOF problem in 3D for a non-maneuvering target. Results of this study shows that these five filters have nearly the same accuracy in operational scenarios. The particle flow filter (PFF), ensemble Kalman filter (EnKF), EKF, UKF, and PF were compared for the AOF problem in 3D for a non-maneuvering target in [37]. It was observed that the EKF-MSC, UKF-MSC, deterministic EnKF-MSC, and Cartesian PFF had the best performance in operational conditions.

In [38], we studied the passive sonar tracking problem when the submarine and the ownship move in different planes using the EKF, UKF, RP-UKF, and PF. Our results showed that the depth of the non-maneuvering target can be estimated accurately, and the PF had the best performance in the scenarios studied. The 3D instrumental variable-based Kalman filter (3D-IVKF) is applied to an underwater passive sonar tracking scenario in [39] for a non-maneuvering target using bearing and elevation measurements. It is observed that at low measurement standard deviations (SDs) (<6°) the performance of the 3D-IVKF is comparable to that of the UKF and CKF. However, at higher measurement SDs, the 3D-IVKF outperforms the UKF and CKF with lower computational cost.

To compare the accuracies of the filters used in the AOF problem with the best achievable accuracy, we computed the posterior Cramér-Rao lower bound (PCRLB) [40] for a non-maneuvering target using the NCV model in [41]. Our results show that when the measurement accuracy is high, the root mean square (RMS) position and velocity errors are close to the corresponding PCRLBs. The difference between RMS position and velocity errors and corresponding PCRLBs increases with a decrease in the measurement accuracy. In [42], a globally valid posterior Cramér–Rao lower bound was derived for the AOF problem. The authors claim the von Mises–Fisher distribution to be superior to the conventional approach using additive Gaussian noise in measured angular coordinates.

A maneuvering target refers to an accelerating target [43]. Common accelerated motions considered in tracking are the nearly constant acceleration (NCA), nearly constant turn (NCT), and jerk [4,22,43]. The NCA and jerk models are linear, whereas the NCT model is nonlinear in the target state. The number of publications for a maneuvering target in the AOF problem is quite limited. In [5], the NCT model was used in the passive ranging problem using an IRST sensor in air-to-air tracking scenarios. The authors used the RP-UKF using the multiple model method. However, algorithm details are not presented in the paper. The NCT model in the XY−plane has been studied extensively where the angular rate is estimated [4,22,43,44,45,46]. This problem arises in the air-traffic control (ATC) scenario [4,22,27,43,47]. In most cases, the conventional discrete-time NCT model is approximate, since the state transition matrix and process noise covariance matrix cannot be derived from the continuous-time model using a consistent procedure.

We consider the tracking of a maneuvering aircraft in 3D and assume that the aircraft moves in the XY-plane with the NCT motion and has a NCV motion along the *Z*-axis. The speed and angular rate are constant for the constant turn motion (CT) in the XY-plane. Thus, it is natural to perturb the speed and angular rate in the NCT motion with the continuous-time white noise (Wiener processes) [22]. We follow this approach from [45] to obtain the nonlinear stochastic differential equation (SDE) [48,49] for the NCT motion. Since the SDE is nonlinear, it cannot be discretized exactly. We discretize the SDE using the first and second-order weak Taylor (TS2) approximations [50] to obtain approximate discrete-time dynamic models. The first-order stochastic Taylor series approximation is also known as the Euler approximation. Two types of states for the NCT motion in the XY-plane, namely the polar velocity and the Cartesian velocity-based states [43,44,45,46], are used. The NCV motion along the *Z*-axis is discretized exactly. The Cartesian velocity state in NCT comprises the 2D position, 2D velocity, and angular rate. In the polar velocity state, the speed and heading replace the 2D Cartesian velocity.

An IRST sensor on another maneuvering aircraft collects azimuth and elevation measurements. The accuracy of the angle measurements by an IRST sensor is usually high (1 mrad). The data rate of an IRST sensor is also high (1 Hz). As sensor technology improves, these factors are expected to improve. The AOF algorithm is required to process the sensor measurements sequentially in real time. Thus, a batch algorithm is ruled out for this tracking scenario. As discussed previously, a PF is not considered for this problem due to its lack of state estimation accuracy and high computational cost. It has been observed in [33,34,35] that when the measurement accuracy and data rate are high (which is true for the current problem), the UKF and CKF have nearly the same accuracy, and the accuracy of the EKF is somewhat lower. If the dimension of the state is *n*, then the UKF and CKF have 2n+1 and 2n sigma points and cubature points, respectively. As a result, the computational cost of the CKF is lower than that of the UKF. If n>3, then the first weight in the UKF becomes negative and the rest of the 2n weights are positive. On the contrary, each of the 2n weights in the CKF is positive and equal to 1/2n. This negative weight may cause a filter-calculated covariance matrix to be non-positive definite in some cases [27]. The CKF was also successfully used in our previous work on AOF in [9]. Hence, we chose the third-degree spherical–radial cubature rule-based CKF [27] to estimate the seven-dimensional state of the maneuvering target. The CKFs using the Euler and TS2 approximations are called CKF1 and CKF2, respectively. Thus, we consider four CKF filters; CKF1P, CKF1C, CKF2P, and CKF2C, where the last letter in the filter refers to polar and Cartesian velocity states.

Notation convention: For clarity, we use italics to denote scalar quantities and boldface for vectors and matrices. A lower or upper-case Roman letter represents a name (e.g., “s” for “sensor,” “RMS” for “root mean square,” etc.). We use “:=” to define a quantity and $A^{\prime }$ denotes the transpose of the vector or matrix A. The n−dimensional identity matrix, m−dimensional null matrix, and m×n null matrix are denoted by $I_{n}$, $0_{m}$, and $0_{m\times n}$, respectively.

The paper is organized as follows. Section 2 presents the dynamic models for the target. Section 3 explains the discretization of target NCT models, and Section 4 describes sensor dynamic and measurement models. A summary of the four CKF filters is given in Section 5. Numerical simulations and results are presented in Section 6. Finally, Section 7 summarizes our contributions in the paper.

## 2. Target Dynamic Models

We assume that the IRST sensor trajectory is deterministic and the states of the sensors are known exactly at measurement times. To improve the observability of the target state, the IRST sensor performs maneuvers with a sequence of CV and constant turn (CT) motions [5,8,31].

Two types of coordinates are commonly used for the NCT in the XY-plane: Cartesian velocity and polar velocity-based models [43,44,45,46]. In addition to the 2D position and velocity, the turn-rate or angular velocity ω is also estimated in the NCT model.

Let z(t) denote the Cartesian state along the *Z*-axis with position and velocity components

(1) $$z\left(t\right):={\left[\begin{array}{cc} z(t) & \dot{z}\left(t\right) \end{array}\right]}^{\prime }.$$

**Figure 1:**
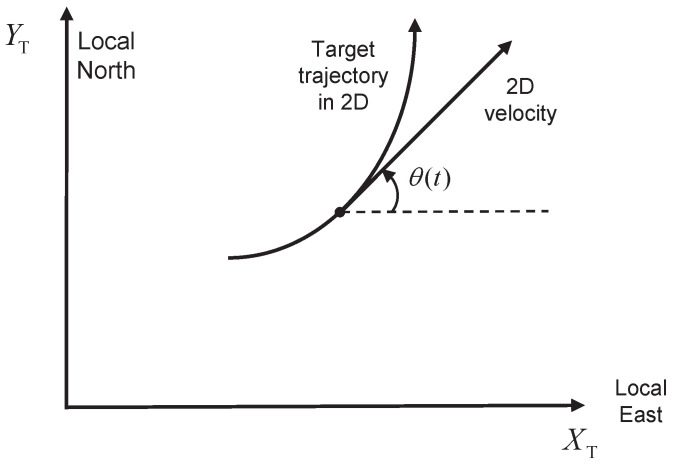
Definition of heading θ(t) in the tracker coordinate frame, θ(t)∈[0,2π).

For the NCT model in the XY-plane, we use η(t) and ξ(t) for state vectors where the angular velocity ω is estimated. The velocity in η(t) and ξ(t) has Cartesian and polar coordinates, respectively. Let s(t) and θ(t) denote the speed and heading of the target in the XY-plane. In this paper, the heading is defined as the angle of the velocity in the XY-plane, measured from the *X*-axis in the counter-clockwise direction, as shown in Figure 1.

Then η(t) and ξ(t) are defined, respectively, by

(2) $$\eta \left(t\right):={\left[\begin{array}{ccccc} x(t) & y(t) & \dot{x}\left(t\right) & \dot{y}\left(t\right) & \omega (t) \end{array}\right]}^{\prime },$$

(3) $$\xi \left(t\right):={\left[\begin{array}{ccccc} x(t) & y(t) & s(t) & \theta (t) & \omega (t) \end{array}\right]}^{\prime }.$$

Three-dimensional state vectors where angular velocity is estimated are defined, respectively, by

(4) $$x_{c}\left(t\right):={\left[\begin{array}{cc} \eta {\left(t\right)}^{\prime } & z{\left(t\right)}^{\prime } \end{array}\right]}^{\prime },$$

(5) $$x_{p}\left(t\right):={\left[\begin{array}{cc} \xi {\left(t\right)}^{\prime } & z{\left(t\right)}^{\prime } \end{array}\right]}^{\prime }.$$

We assume that the measurement time interval is constant; i.e., $t_{k}-t_{k-1}=T$ for all *k*. In this paper, we use the discretized continuous-time models [22].

The discrete-time dynamic model for the NCV motion along the *Z*-axis is given by

(6) $$z_{k}=F_{1}z_{k-1}+w_{z,k-1},$$

where $F_{1}$ is the state transition matrix and $w_{z,k-1}$ is a zero-mean white Gaussian process noise with covariance $Q_{z}$,

(7) $$F_{1}=\left[\begin{array}{cc} 1 & T\\ 0 & 1 \end{array}\right],$$

(8) $$Q_{z}=q_{z}B,$$

(9) $$B=\left[\begin{array}{cc} T^{3}/3 & T^{2}/2\\ T^{2}/2 & T \end{array}\right],$$

where $q_{z}$ is the power spectral density (PSD) of the continuous-time acceleration process noise along the *Z*-axis [22].

The time derivative of ξ(t) is given by

(10) $$\dot{\xi }\left(t\right)={\left[\begin{array}{ccccc} \dot{x}\left(t\right) & \dot{y}\left(t\right) & \dot{s}\left(t\right) & \dot{\theta }\left(t\right) & \dot{\omega }\left(t\right) \end{array}\right]}^{\prime }.$$

We have

(11) $$\dot{x}\left(t\right)=s\left(t\right)\cos\theta \left(t\right),\;\dot{y}\left(t\right)=s\left(t\right)\sin\theta \left(t\right).$$

Since $\dot{\theta }\left(t\right)=\omega \left(t\right)$, (10) can be written as

(12) $$\dot{\xi }\left(t\right)={\left[\begin{array}{ccccc} s(t)\cos\theta (t) & s(t)\sin\theta (t) & \dot{s}\left(t\right) & \omega (t) & \dot{\omega }\left(t\right) \end{array}\right]}^{\prime }.$$

The time derivative of η(t) is

(13) $$\dot{\eta }\left(t\right)={\left[\begin{array}{ccccc} \dot{x}\left(t\right) & \dot{y}\left(t\right) & \ddot{x}\left(t\right) & \ddot{y}\left(t\right) & \dot{\omega }\left(t\right) \end{array}\right]}^{\prime }.$$

In the constant turn (CT) model, the speed and turn rate are constant. The speed and turn rate can be modeled as nearly constant in the NCT motion. Examining (12) and (13), we find that for the NCT model, (12) is more suitable than (13), based on symmetry considerations. Using conventional models in the engineering literature [22], for the NCT model, we may write

(14) $$\dot{s}\left(t\right)=w_{s}\left(t\right),\;\dot{\omega }\left(t\right)=w_{\omega }\left(t\right),$$

where $w_{s}\left(t\right)$ and $w_{\omega }\left(t\right)$ are continuous-time zero-mean white acceleration and angular acceleration process noises with power spectral densities $q_{s}$ and $q_{\omega },$ respectively, [22]

(15) $$E\left\{w_{s}\left(t\right)\right\}=0,\;E\left\{w_{s}\left(t\right)w_{s}\left(\tau \right)\right\}=q_{s}\delta \left(t-\tau \right),$$

(16) $$E\left\{w_{\omega }\left(t\right)\right\}=0,\;E\left\{w_{\omega }\left(t\right)w_{\omega }\left(\tau \right)\right\}=q_{\omega }\delta \left(t-\tau \right),$$

where δ is the Dirac delta function [51]. We can write (14)–(16) mathematically rigorously by defining

(17) $$ds\left(t\right)=\sqrt{q_{s}}d\beta_{s}\left(t\right),\;d\omega \left(t\right)=\sqrt{q_{\omega }}d\beta_{\omega }\left(t\right),$$

where $d\beta_{s}\left(t\right)$ and $d\beta_{\omega }\left(t\right)$ are standard independent Wiener processes [45,48]

(18) $$E\left\{d\beta_{s}\left(t\right)d\beta_{s}\left(t\right)\right\}=dt,\;E\left\{d\beta_{\omega }\left(t\right)d\beta_{\omega }\left(t\right)\right\}=dt,$$

(19) $$E\{d\beta_{s}\left(t\right)d\beta_{\omega }\left(t\right)\}=0.$$

Define

(20) $$f_{p}\left(\xi \left(t\right)\right):={\left[\begin{array}{ccccc} s(t)\cos\theta (t) & s(t)\sin\theta (t) & 0 & \omega (t) & 0 \end{array}\right]}^{\prime },$$

(21) $$w_{p}\left(t\right):={\left[\begin{array}{ccccc} 0 & 0 & w_{s}\left(t\right) & 0 & w_{\omega }\left(t\right) \end{array}\right]}^{\prime },$$

(22) $$G_{p}:={\left[\begin{array}{ccccc} 0 & 0 & \sqrt{q_{s}} & 0 & 0\\ 0 & 0 & 0 & 0 & \sqrt{q_{\omega }} \end{array}\right]}^{\prime },$$

(23) $$d\beta \left(t\right):={\left[\begin{array}{cc} d\beta_{s}\left(t\right) & d\beta_{\omega }\left(t\right) \end{array}\right]}^{\prime }.$$

Then, conventionally, we can write $\dot{\xi }\left(t\right)$ as [23,48]

(24) $$\dot{\xi }\left(t\right)=f_{p}\left(\xi \left(t\right)\right)+w_{p}\left(t\right).$$

We can write the time derivative of the polar state vector mathematically rigorously using the Itô stochastic differential equation (SDE) [45,48,49]

(25) $$d\xi \left(t\right)=f_{p}\left(\xi \left(t\right)\right)dt+G_{p}d\beta \left(t\right),$$

where

(26) $$E\left\{d\beta \left(t\right)d\beta^{\prime }\left(t\right)\right\}=I_{2}dt.$$

We assume that the prior distribution of ξ is Gaussian,

(27) $$\xi_{0}=\xi \left(t_{0}\right)∼N\left(\xi_{0};{\bar{\xi }}_{0},P_{0}^{\xi }\right).$$

The time derivative of η contains Cartesian accelerations $\ddot{x}$ and $\ddot{y}$ in (13). It is necessary to transform them to derivatives of speed and angular velocity. The 2D Cartesian velocity is given by

(28) $$v\left(t\right)={\left[\begin{array}{cc} \dot{x}\left(t\right) & \dot{y}\left(t\right) \end{array}\right]}^{\prime }$$

and the Cartesian acceleration is $\dot{v}\left(t\right).$ Using (11), the Cartesian acceleration is expressed by

(29) $$\dot{v}\left(t\right)={\left[\begin{array}{cc} \dot{s}\left(t\right)\cos\theta \left(t\right)-\omega \left(t\right)\dot{y}\left(t\right) & \dot{s}\left(t\right)\sin\theta \left(t\right)+\omega \left(t\right)\dot{x}\left(t\right) \end{array}\right]}^{\prime }.$$

Using a similar approach, the Itô SDE [45,48,49] for the Cartesian state is

(30) $$d\eta \left(t\right)=f_{c}\left(\eta \left(t\right)\right)dt+G_{c}\left(\eta \left(t\right)\right)d\beta \left(t\right),$$

where

(31) $$f_{c}\left(\eta \left(t\right)\right):={\left[\begin{array}{ccccc} \dot{x}\left(t\right) & \dot{y}\left(t\right) & -\omega \left(t\right)\dot{y}\left(t\right) & \omega \left(t\right)\dot{x}\left(t\right) & 0 \end{array}\right]}^{\prime },$$

(32) $$G_{c}\left(\eta \left(t\right)\right):={\left[\begin{array}{ccccc} 0 & 0 & \sqrt{q_{s}}\dot{x}\left(t\right)/s\left(t\right) & \sqrt{q_{s}}\dot{y}\left(t\right)/s\left(t\right) & 0\\ 0 & 0 & 0 & 0 & \sqrt{q_{\omega }} \end{array}\right]}^{\prime }.$$

## 3. Discretization of Target NCT Models

### 3.1. The Euler Approximation

The Euler approximation is obtained by applying the Itô lemma [48] to the integral form of the SDE and retaining only single integral terms. Applying the Euler approximation [50] to the 2D polar velocity dynamic model, we obtain the stochastic difference equation [45]:

(33) $$\xi_{k}=\xi_{k-1}+Tf_{p}\left(\xi_{k-1}\right)+\sqrt{T}G_{p}w_{1},$$

where $f_{p}\left(\xi \right)$ is defined in (20) and

(34) $$w_{1}∼N\left(w_{1};0_{2\times 1},I_{2}\right).$$

The covariance of the polar velocity process noise $w_{k-1}^{\xi }=\sqrt{T}G_{p}w_{1}$ is described by

(35) $$w_{k-1}^{\xi }∼N\left(w_{k-1}^{\xi };0_{5\times 1},Q^{\xi }\right),$$

(36) $$Q^{\xi }=\mathrm{diag}\left(0,0,q_{s}T,0,q_{\omega }T\right).$$

From (36), we see that the polar velocity process noise is independent of the state.

Similarly, applying the Euler approximation to the Cartesian velocity dynamic model, we obtain the stochastic difference equation [45]

(37) $$\eta_{k}=\eta_{k-1}+Tf_{c}\left(\eta_{k-1}\right)+\sqrt{T}G_{c}\left(\eta_{k-1}\right)w_{1},$$

where $f_{c}\left(\eta \right)$ is defined in (31). The Cartesian velocity process noise $w_{k-1}^{\eta }=\sqrt{T}G_{c}\left(\eta_{k-1}\right)w_{1}$ is described by

(38) $$w_{k-1}^{\eta }\left(\eta_{k-1}\right)∼N\left(w_{k-1}^{\eta };0_{5\times 1},Q_{k-1}^{\eta }\right),$$

(39) $$Q_{k-1}^{\eta }=TE\left\{G_{c}\left(\eta_{k-1}\right)G_{c}^{\prime }\left(\eta_{k-1}\right)\right\}.$$

We make the following approximation in calculating $E\{G_{c}\left(\eta_{k-1}\right)G_{c}^{\prime }\left(\eta_{k-1}\right)\}$,

(40) $$E\left\{G_{c}\left(\eta_{k-1}\right)G_{c}^{\prime }\left(\eta_{k-1}\right)\right\}\approx G_{c}\left({\hat{\eta }}_{k|k-1}\right)G_{c}^{\prime }\left({\hat{\eta }}_{k|k-1}\right),$$

where ${\hat{\eta }}_{k|k-1}$ is the predicted Cartesian velocity state estimate at time *k*. Then,

(41) $$Q_{k-1}^{\eta }\approx TG_{c}\left({\hat{\eta }}_{k|k-1}\right)G_{c}^{\prime }\left({\hat{\eta }}_{k|k-1}\right).$$

Simplification of (41) gives

(42) $$Q_{k-1}^{\eta }\approx {\left[\begin{array}{ccc} 0_{2} & 0_{2} & 0_{2\times 1}\\ 0_{2} & q_{s}TA_{k-1}\left({\hat{\eta }}_{k|k-1}\right) & 0_{2\times 1}\\ 0_{1\times 2} & 0_{1\times 2} & q_{\omega }T \end{array}\right]}^{\prime },$$

where

(43) $$A_{k-1}\left({\hat{\eta }}_{k|k-1}\right)=\frac{1}{{\hat{s}}_{k|k-1}^{2}}{\left[\begin{array}{cc} {\hat{\dot{x}}}_{k|k-1}^{2} & {\hat{\dot{x}}}_{k|k-1}{\hat{\dot{y}}}_{k|k-1}\\ {\hat{\dot{x}}}_{k|k-1}{\hat{\dot{y}}}_{k|k-1} & {\hat{\dot{y}}}_{k|k-1}^{2} \end{array}\right]}^{\prime }.$$

From (42) and (43), we see that the Cartesian velocity-based process noise covariance is state-dependent.

From (4) and (5), we get the polar and Cartesian velocity-based states as

(44) $$x_{p,k}={\left[\begin{array}{cc} \xi_{k}^{\prime } & z_{k}^{\prime } \end{array}\right]}^{\prime },$$

(45) $$x_{c,k}={\left[\begin{array}{cc} \eta_{k}^{\prime } & z_{k}^{\prime } \end{array}\right]}^{\prime }.$$

The 3D discrete-time dynamic model for the polar velocity-based model is given by

(46) $$x_{p,k}=x_{p,k-1}+T{\tilde{f}}_{p}\left(x_{p,k-1}\right)+w_{p,k-1},$$

where ${\tilde{f}}_{p}\left(x_{p}\right)$ is defined by

(47) $${\tilde{f}}_{p}\left(x_{p}\right)={\left[\begin{array}{ccccccc} s\cos\theta & s\sin\theta & 0 & \omega & 0 & \dot{z} & 0 \end{array}\right]}^{\prime },$$

(48) $$w_{p,k-1}:={\left[\begin{array}{cc} {\left(w_{k-1}^{\xi }\right)}^{\prime } & w_{z,k-1}^{\prime } \end{array}\right]}^{\prime },$$

(49) $$w_{p,k-1}∼N\left(w_{p,k-1};0_{7\times 1},Q_{p}\right),$$

(50) $$Q_{p}=\left[\begin{array}{cc} Q^{\xi } & 0_{5\times 2}\;\\ 0_{2\times 5} & Q_{z} \end{array}\right].$$

Similarly, the 3D discrete-time dynamic model for the Cartesian velocity-based model is given by

(51) $$x_{c,k}=x_{c,k-1}+T{\tilde{f}}_{c}\left(x_{c,k-1}\right)+w_{c,k-1},$$

where ${\tilde{f}}_{c}\left(x_{c}\right)$ is defined by

(52) $${\tilde{f}}_{c}\left(x_{c}\right)={\left[\begin{array}{ccccccc} \dot{x} & \dot{y} & -\omega \dot{y} & \omega \dot{x} & 0 & \dot{z} & 0 \end{array}\right]}^{\prime },$$

(53) $$w_{c,k-1}:={\left[\begin{array}{cc} {\left(w_{k-1}^{\eta }\right)}^{\prime } & w_{z,k-1}^{\prime } \end{array}\right]}^{\prime },$$

(54) $$w_{c,k-1}∼N\left(w_{c,k-1};0_{7\times 1},Q_{c,k-1}\right),$$

(55) $$Q_{c,k-1}=\left[\begin{array}{cc} Q_{k-1}^{\eta } & 0_{5\times 2}\;\\ 0_{2\times 5} & Q_{z} \end{array}\right].$$

### 3.2. Order 2 Weak Taylor Approximation

Using the order 2 weak Taylor approximation [50] to the SDE, we obtain the discretized dynamic model for the polar velocity-based model as [45]

(56) $$\xi_{k}=\xi_{k-1}+Tf_{p,2}\left(\xi_{k-1}\right)+G_{p,2}\left(\xi_{k-1}\right)w_{2},$$

where

(57) $$f_{p,2}\left(\xi_{k-1}\right)=\left[\begin{array}{c} s_{k-1}\cos\left(\theta_{k-1}\right)-Ts_{k-1}\omega_{k-1}\sin\left(\theta_{k-1}\right)/2\\ s_{k-1}\sin\left(\theta_{k-1}\right)+Ts_{k-1}\omega_{k-1}\cos\left(\theta_{k-1}\right)/2\\ 0\\ \omega_{k-1}\\ 0 \end{array}\right],$$

(58) $$G_{p,2}\left(\xi_{k-1}\right)=E_{p}\left(\xi_{k-1}\right)V\left(T\right),$$

(59) $$E_{p}\left(\xi_{k-1}\right)=\left[\begin{array}{cccc} \sqrt{q_{s}}\cos\left(\theta_{k-1}\right) & 0 & 0 & 0\\ \sqrt{q_{s}}\sin\left(\theta_{k-1}\right) & 0 & 0 & 0\\ 0 & 0 & \sqrt{q_{s}} & 0\\ 0 & \sqrt{q_{\omega }} & 0 & 0\\ 0 & 0 & 0 & \sqrt{q_{\omega }} \end{array}\right],$$

(60) $$V\left(T\right)=\left[\begin{array}{cc} \sqrt{T^{3}/3} & 0\\ \sqrt{3T}/2 & \sqrt{T}/2 \end{array}\right]⊗I_{2},$$

(61) $$w_{2}∼N\left(w_{2};0_{4\times 1},I_{4}\right).$$

In (60), ⊗ refers to the Kronecker product [52].

The process noise $w_{p,2,k-1}=G_{p,2}\left(\xi_{k-1}\right)w_{2}$ and associated covariance $Q_{p,2,k-1}$ for the second-order polar velocity-based model are described, respectively, by

(62) $$w_{p,2,k-1}∼N\left(w_{p,2,k-1};0_{5\times 1},Q_{p,2,k-1}\right),$$

(63) $$Q_{p,2,k-1}=E\left\{G_{p,2}\left(\xi_{k-1}\right)G_{p,2}^{\prime }\left(\xi_{k-1}\right)\right\}.$$

Using a similar approximation as before, we obtain

(64) $$Q_{p,2,k-1}\approx G_{p,2}\left({\hat{\xi }}_{k|k-1}\right)G_{p,2}^{\prime }\left({\hat{\xi }}_{k|k-1}\right),$$

where ${\hat{\xi }}_{k|k-1}$ is the predicted polar velocity state estimate at time k.

Simplification of $G_{p,2}\left(\xi_{k-1}\right)G_{p,2}^{\prime }\left(\xi_{k-1}\right)$ gives

(65) $$G_{p,2}\left(\xi_{k-1}\right)G_{p,2}^{\prime }\left(\xi_{k-1}\right)=\left[\begin{array}{ccccc} \frac{T^{3}\cos^{2}\left(\theta_{k-1}\right)}{3}q_{s} & \frac{T^{3}\sin\left(2\theta_{k-1}\right)}{6}q_{s}\; & \frac{T^{2}\cos\left(\theta_{k-1}\right)}{2}q_{s} & 0 & 0\;\\ \frac{T^{3}\sin\left(2\theta_{k-1}\right)}{6}q_{s} & \frac{T^{3}\sin^{2}\left(\theta_{k-1}\right)}{3}q_{s}\; & \frac{T^{2}\sin\left(\theta_{k-1}\right)}{2}q_{s} & 0 & 0\;\\ \frac{T^{2}\cos\left(\theta_{k-1}\right)}{2}q_{s} & \frac{T^{2}\sin\left(\theta_{k-1}\right)}{2}q_{s} & Tq_{s} & 0 & 0\;\\ 0 & 0 & 0 & \frac{T^{3}}{3}q_{\omega } & \frac{T^{2}}{2}q_{\omega }\;\\ 0 & 0 & 0 & \frac{T^{2}}{2}q_{\omega } & Tq_{\omega } \end{array}\right].$$

The discretized dynamic model for the Cartesian velocity-based model using the TS2 approximation to the SDE is given by [45]

(66) $$\eta_{k}=\eta_{k-1}+Tf_{c,2}\left(\eta_{k-1}\right)+G_{c,2}\left(\eta_{k-1}\right)w_{2},$$

where

(67) $$f_{c,2}\left(\eta_{k-1}\right)=\left[\begin{array}{c} {\dot{x}}_{k-1}-T\omega_{k-1}{\dot{y}}_{k-1}/2\\ {\dot{y}}_{k-1}+T\omega_{k-1}{\dot{x}}_{k-1}/2\\ -\omega_{k-1}{\dot{y}}_{k-1}-T\omega_{k-1}^{2}{\dot{x}}_{k-1}/2\\ \omega_{k-1}{\dot{x}}_{k-1}-T\omega_{k-1}^{2}{\dot{y}}_{k-1}/2\\ 0 \end{array}\right],$$

(68) $$G_{c,2}\left(\eta_{k-1}\right)=E_{c}\left(\eta_{k-1}\right)V\left(T\right),$$

(69) $$E_{c}\left(\eta_{k-1}\right)=\left[\begin{array}{cccc} \sqrt{q_{s}}{\dot{x}}_{k-1}/s_{k-1} & 0 & 0 & 0\\ \sqrt{q_{s}}{\dot{y}}_{k-1}/s_{k-1} & 0 & 0 & 0\\ 0 & -\sqrt{q_{\omega }}{\dot{y}}_{k-1} & \sqrt{q_{s}}\left({\dot{x}}_{k-1}-T\omega_{k-1}{\dot{y}}_{k-1}\right)/s_{k-1} & 0\\ 0 & \sqrt{q_{\omega }}{\dot{x}}_{k-1} & \sqrt{q_{s}}\left({\dot{y}}_{k-1}+T\omega_{k-1}{\dot{x}}_{k-1}\right)/s_{k-1} & 0\\ 0 & 0 & 0 & \sqrt{q_{\omega }} \end{array}\right].$$

The process noise $w_{c,2,k-1}=G_{c,2}\left(\eta_{k-1}\right)w_{2}$, and corresponding covariance $Q_{c,2,k-1}$ for the second-order Cartesian velocity-based model are given, respectively, by

(70) $$w_{c,2,k-1}∼N\left(w_{c,2,k-1};0_{5\times 1},Q_{c,2,k-1}\right),$$

(71) $$Q_{c,2,k-1}=E\left\{G_{c,2}\left(\eta_{k-1}\right)G_{c,2}^{\prime }\left(\eta_{k-1}\right)\right\}.$$

The approximate expression for the process noise is given by

(72) $$Q_{c,2,k-1}\approx G_{c,2}\left({\hat{\eta }}_{k|k-1}\right)G_{c,2}^{\prime }\left({\hat{\eta }}_{k|k-1}\right),$$

where ${\hat{\eta }}_{k|k-1}$ is the predicted state estimate at time k. Simplification of $G_{c,2}\left(\eta_{k-1}\right)G_{c,2}^{\prime }\left(\eta_{k-1}\right)$ gives

(73) $$\begin{array}{cc} \; & G_{c,2}\left(\eta_{k-1}\right)G_{c,2}^{\prime }\left(\eta_{k-1}\right)=\\ \; & \left[\begin{array}{ccccc} \frac{T^{3}}{3}a_{1}^{2}q_{s} & \frac{T^{3}}{3}a_{1}a_{2}q_{s} & \frac{T^{2}}{2}a_{1}a_{3}q_{s} & \frac{T^{2}}{2}a_{1}a_{4}q_{s} & 0\\ \frac{T^{3}}{3}a_{1}a_{2}q_{s} & \frac{T^{3}}{3}a_{2}^{2}q_{s} & \frac{T^{2}}{2}a_{2}a_{3}q_{s}\; & \frac{T^{2}}{2}a_{2}a_{4}q_{s} & 0\\ \frac{T^{2}}{2}a_{1}a_{3}q_{s} & \frac{T^{2}}{2}a_{2}a_{3}q_{s} & Ta_{3}^{2}q_{s}+\frac{T^{3}}{3}{\dot{y}}_{k-1}^{2}q_{\omega }\; & Ta_{3}a_{4}q_{s}-\frac{T^{3}}{3}{\dot{x}}_{k-1}{\dot{y}}_{k-1}q_{\omega } & -\frac{T^{2}}{2}{\dot{y}}_{k-1}q_{\omega }\\ \frac{T^{2}}{2}a_{1}a_{4}q_{s} & \frac{T^{2}}{2}a_{2}a_{4}q_{s} & Ta_{3}a_{4}q_{s}-\frac{T^{3}}{3}{\dot{x}}_{k-1}{\dot{y}}_{k-1}q_{\omega }\; & Ta_{4}^{2}q_{s}+\frac{T^{3}}{3}{\dot{x}}_{k-1}^{2}q_{\omega } & \frac{T^{2}}{2}{\dot{x}}_{k-1}q_{\omega }\\ 0 & 0 & -\frac{T^{2}}{2}{\dot{y}}_{k-1}q_{\omega } & \frac{T^{2}}{2}{\dot{x}}_{k-1}q_{\omega } & Tq_{\omega } \end{array}\right], \end{array}$$

where

(74) $$a_{1}=\frac{{\dot{x}}_{k-1}}{s_{k-1}},\;a_{2}=\frac{{\dot{y}}_{k-1}}{s_{k-1}},$$

(75) $$a_{3}=\frac{{\dot{x}}_{k-1}-T\omega_{k-1}{\dot{y}}_{k-1}}{s_{k-1}},\;a_{4}=\frac{{\dot{y}}_{k-1}+T\omega_{k-1}{\dot{x}}_{k-1}}{s_{k-1}}.$$

### 3.3. Comparison with Conventional NCT Model

We consider the NCT model using the Cartesian velocity-based state, when the angular rate is estimated. The NCT model using the direct discrete approach is described in Chapter 11 of [22]. The discretized continuous-time NCT model [27] is described by

(76) $$\eta_{k}=F_{\mathrm{NCT}}^{C}\left(\omega \right)\eta_{k-1}+w_{k-1}^{C},$$

(77) $$F_{\mathrm{NCT}}^{C}\left(\omega \right)=\left[\begin{array}{ccccc} 1 & 0 & \frac{\sin(\omega T)}{\omega } & -\frac{1-\cos(\omega T)}{\omega } & 0\\ 0 & 1 & \frac{1-\cos(\omega T)}{\omega } & \frac{\sin(\omega T)}{\omega } & 0\\ 0 & 0 & \cos(\omega T) & -\sin(\omega T) & 0\\ 0 & 0 & \sin(\omega T) & \cos(\omega T) & 0\\ 0 & 0 & 0 & 0 & 1 \end{array}\right],$$

(78) $$w_{k-1}^{C}∼N\left(w_{k-1}^{C};0,Q_{k-1}^{C}\right),$$

(79) $$Q_{k-1}^{C}=\left[\begin{array}{ccccc} qT^{3}/3 & 0 & qT^{2}/2 & 0 & 0\\ 0 & qT^{3}/3 & 0 & qT^{2}/2 & 0\\ qT^{2}/2 & 0 & qT & 0 & 0\\ 0 & qT^{2}/2 & 0 & qT & 0\\ 0 & 0 & 0 & 0 & q_{\omega }T \end{array}\right],$$

where *q* is the PSD of the acceleration process noise along the *X* or *Y* direction.

**Remark** **1.** *The upper left 4×4 block of the state transition matrix in (77) is the state transition matrix for the NCV model using Cartesian state [22]. Similarly, the upper left 4×4 block of the process noise covariance matrix in (79) is the process noise covariance matrix for the NCV model using Cartesian state [22]. They cannot be derived from a continuous-time model of the NCT motion.*

The second-order model with the TS2 approximation and Cartesian velocity-based state was used in [53], and a superior RMSE was reported compared with the conventional model described above.

## 4. Sensor Dynamic and Measurement Models

### 4.1. Sensor Dynamic Models

We assume that the motion of the sensor is deterministic and the state of the sensor at each measurement time is exactly known. The sensor follows two types of motion: constant velocity (CV) in 3D and the second type of motion with known angular velocity Ω. For both types of motion, the Cartesian state vector of the sensor is appropriate and is defined by

(80) $$x^{s}\left(t\right):={\left[\begin{array}{cccccc} x^{s}\left(t\right) & y^{s}\left(t\right) & {\dot{x}}^{s}\left(t\right) & {\dot{y}}^{s}\left(t\right) & z^{s}\left(t\right) & {\dot{z}}^{s}\left(t\right) \end{array}\right]}^{\prime }.$$

The dynamic models of the sensor for the CV and CT are described, respectively, by [8,31]

(81) $$x_{k}^{s}=F^{\mathrm{CV}}\left(T\right)x_{k-1}^{s},$$

(82) $$F^{\mathrm{CV}}\left(T\right)=\left[\begin{array}{ccc} I_{2} & I_{2}T & 0_{2}\\ 0_{2} & I_{2} & 0_{2}\\ 0_{2} & 0_{2} & F_{1} \end{array}\right].$$

(83) $$x_{k}^{s}=F^{\mathrm{CT}}\left(T,\Omega_{k-1}\right)x_{k-1}^{s},$$

where the state transition matrix $F_{1}$ for CV is defined in (7), $\Omega_{k-1}$ is the angular velocity of the sensor during $\left[t_{k-1},t_{k}\right)$ and the state transition matrix for CT is given by

(84) $$F^{\mathrm{CT}}\left(T,\Omega \right)=\left[\begin{array}{cccccc} 1 & 0 & \sin(\Omega T)/\Omega & -[1-\cos(\Omega T)]/\Omega & 0 & 0\\ 0 & 1 & [1-\cos(\Omega T)]/\Omega & \sin(\Omega T)/\Omega & 0 & 0\\ 0 & 0 & \cos(\Omega T) & -\sin(\Omega T) & 0 & 0\\ 0 & 0 & \sin(\Omega T) & \cos(\Omega T) & 0 & 0\\ 0 & 0 & 0 & 0 & 1 & T\\ 0 & 0 & 0 & 0 & 0 & 1 \end{array}\right].$$

In passive IRST tracking, the sensor moves with a sequence of CV and CT motions [8,31].

### 4.2. Measurement Model

Let $p_{k}$ and $p_{k}^{s}$ denote the target and sensor position vectors, respectively, at time $t_{k}$,

(85) $$p_{k}:={\left[x_{k}\;y_{k}\;z_{k}\right]}^{\prime },$$

(86) $$p_{k}^{s}:={\left[x_{k}^{s}\;y_{k}^{s}\;z_{k}^{s}\right]}^{\prime }.$$

**Figure 2:**
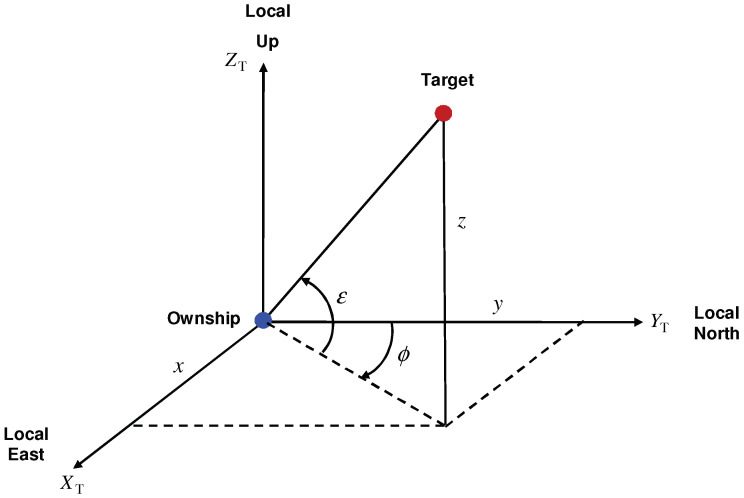
Definition of the tracker coordinate frame (T frame), bearing ϕ∈[0,2π) and elevation ϵ∈(−π/2,π/2).

An IRST sensor measures the bearing and elevation angles of a target [5,8], as shown in Figure 2. We note that the bearing ($\phi_{k}$) and elevation ($\epsilon_{k}$) angles depend on the relative position $p_{k}-p_{k}^{s}$ in Cartesian and polar velocity-based models. Hence, for both type of state vectors, the measurement model for the bearing and elevation angles is described by

(87) $$y_{k}=h\left(p_{k},p_{k}^{s}\right)+n_{k},$$

(88) $$h\left(p_{k},p_{k}^{s}\right):=\left[\begin{array}{c} \phi_{k}\\ \epsilon_{k} \end{array}\right]=\left[\begin{array}{c} \tan^{-1}\left(x_{k}-x_{k}^{s},y_{k}-y_{k}^{s}\right)\\ \arctan(\left(z_{k}-z_{k}^{s}\right)/\rho_{k}) \end{array}\right],$$

where $\phi_{k}$ and $\epsilon_{k}$ lie in [0,2π) and (−π/2,π/2), respectively and the ground range $\rho_{k}$ is defined by

(89) $$\rho_{k}:=\sqrt{{\left(x_{k}-x_{k}^{s}\right)}^{2}+{\left(y_{k}-y_{k}^{s}\right)}^{2}},\;\rho_{k}>0.$$

We assume that the measurement noise is zero-mean Gaussian with covariance R

(90) $$n_{k}∼N\left(n_{k};0,R\right),$$

(91) $$R:=\mathrm{diag}(\sigma_{\phi }^{2},\sigma_{\epsilon }^{2}),$$

where $\sigma_{\phi }$ and $\sigma_{\epsilon }$ are the bearing and elevation measurement standard deviations (SDs), respectively.

## 5. Filtering Algorithms

We compare the performances of four CKF-based algorithms using the Euler and TS2 approximations with the polar and Cartesian velocity-based states. These four algorithms are called CKF1P, CKF1C, CKF2P, and CKF1P. The discrete-time dynamic and measurement models in these algorithms are nonlinear. The features of these algorithms are summarized in Table 1. In [54], the authors have considered the maneuvering target tracking problem using a CKF-based CDF filter with range, azimuth, and elevation measurements. They claim that this is a very challenging problem. They use the prior distribution to initialize the filter. The problem considered in our study is relatively harder since only azimuth, and elevation measurements are available.

## 6. Numerical Simulation and Results

The IRST sensor trajectory and parameters in the simulation are similar to those used in [8,31]. The target moves with an NCT motion in a plane parallel to the XY-plane and moves with an NCV motion along the *Z*-axis. Table 2 presents prior mean polar velocity-based state parameters of the target. The NCT motion has a centripetal acceleration $s_{1}\omega_{1}$ of 3 g, where $g=9.8\;m^{2}s^{-2}$. This scenario was used in [5]. We use the same filter initialization with that in [54] in the current study. The prior variance of the 3D polar velocity-based state is chosen as

(92) $$\begin{array}{cc} P_{0,p,1} & =\mathrm{diag}(1000^{2}\;m^{2},\;1000^{2}\;m^{2},\;30^{2}\;m^{2}s^{-2},\;0.0873\;{\mathrm{rad}}^{2},\;{\left(4.95\times 10^{-3}\right)}^{2}\;{\mathrm{rad}}^{2}s^{-2},\\ \; & \;100^{2}\;m^{2},5^{2}\;m^{2}s^{-2}). \end{array}$$

**Figure 3:**
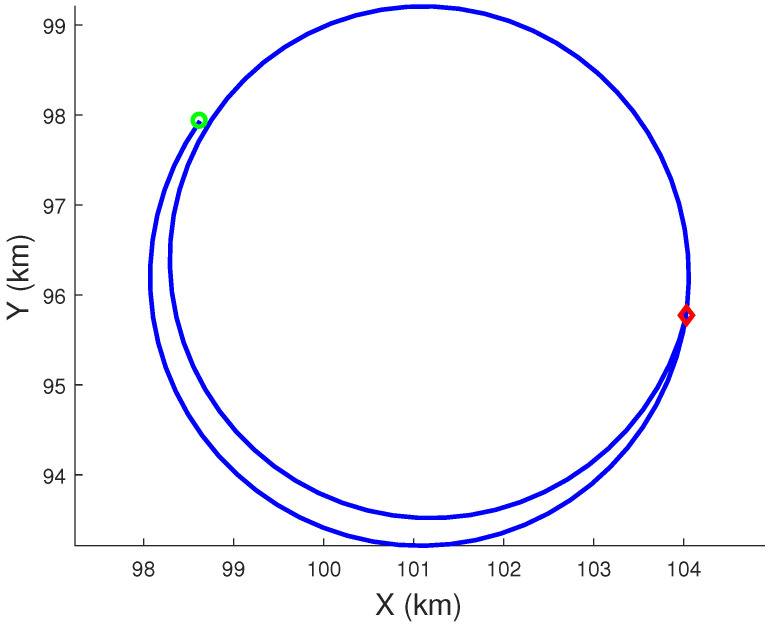
Target true trajectory in the XY-plane from the first Monte Carlo run. The green circle and the red diamond represent the start point and end point, respectively.

Using the Euler approximation, the process noise covariance in the polar velocity-based model for the NCT motion can be calculated exactly. Hence, we use the Euler approximation for the polar velocity-based model to generate true target trajectories for the NCT motion in the XY-plane using 100 sub-sampling intervals for the measurement time interval (*T*) of 1 s. The *Z*-component of the NCV trajectory is generated exactly. The process noise parameters used in the simulation are $q_{s}=0.2\;m^{2}s^{-3}$, $q_{\omega }=5e-07\;{\mathrm{rad}}^{2}s^{-3}$, and $q_{z}=0.001\;m^{2}s^{-3}.$ Figure 3 presents the true NCT trajectory of the target in the XY-plane from the first Monte Carlo run.

**Figure 4:**
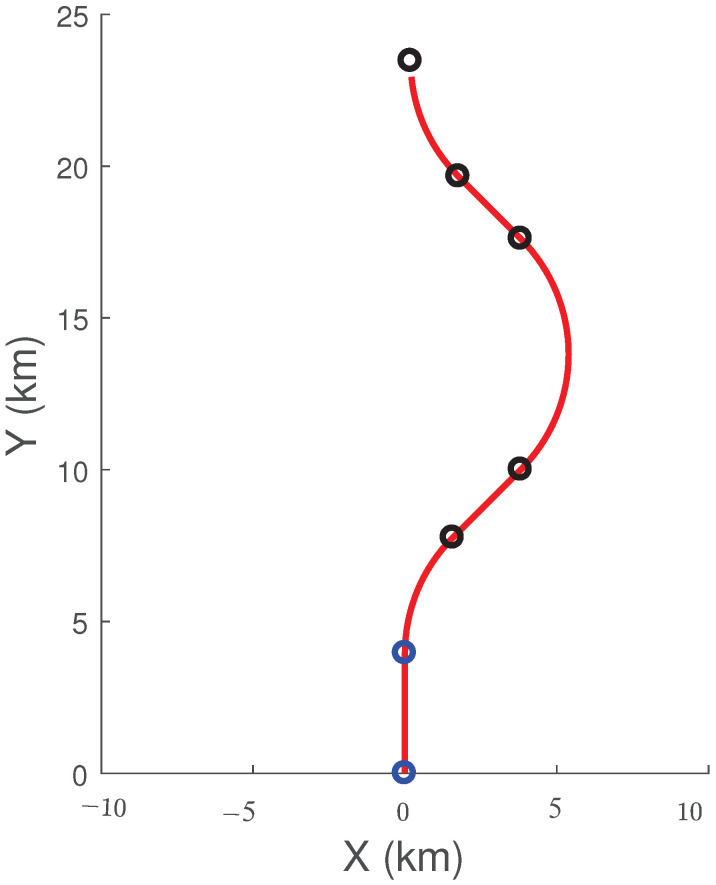
Sensor trajectory in the XY-plane.

We assume that the motion of the sensor is deterministic. The sensor moves in a plane parallel to the XY-plane at a fixed height of 10 km and follows a sequence of CV and CT motions. The initial position and velocity vectors of the sensor are (0, 0, 10,000) m and (0, 264, 0) m/s, respectively. Table 3 presents the motion profile of the sensor. In Table 3, Δt represents the duration of the segment, $\Delta \phi_{s}$ is the total angular change during the segment, and Ω is the angular velocity of the sensor during the segment. The measurement time interval of the IRST sensor is 1 s and there are 101 measurements. The measurement error SDs for bearing and elevation have the same value. We use two angle SDs of 1 mrad and 2 mrad in this simulation. The sensor trajectory in the XY-plane is shown in Figure 4.

### 6.1. Comparison of Filtering Algorithms

**Figure 5:**
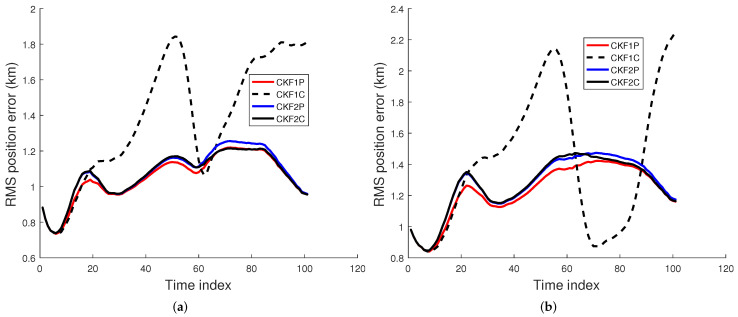
RMS position error using the prior variance $P_{0,p,1}$ from 500 Monte Carlo runs. (**a**) Angle SD of 1 mrad, (**b**) Angle SD of 2 mrad.

**Figure 6:**
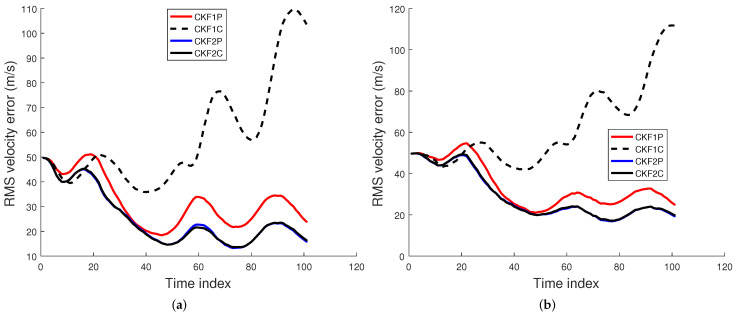
RMS velocity error using the prior variance $P_{0,p,1}$ from 500 Monte Carlo runs. (**a**) Angle SD of 1 mrad, (**b**) Angle SD of 2 mrad.

**Figure 7:**
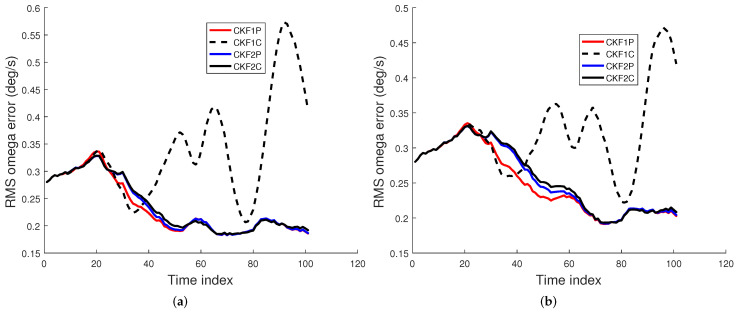
RMS angular rate error using the prior variance $P_{0,p,1}$ from 500 Monte Carlo runs. (**a**) Angle SD of 1 mrad, (**b**) Angle SD of 2 mrad.

We used 500 Monte Carlo runs to compute the root mean square (RMS) position, velocity, and angular rate errors of the CKF1P, CKF1C, CKF2P, and CKF2C. Each filter is initialized using the prior mean and covariance. The RMS errors for these four filters for angle SDs of 1 mrad and 2 mrad are presented in Figure 5, Figure 6 and Figure 7. Results in Figure 5 show that RMS position errors of the CKF1P, CKF2P, and CKF2C are close and they are nearly the same towards the end. On the contrary, the RMS position error of the CKF1C is significantly higher during some measurement intervals and also significantly lower during a time interval. We see in Figure 6 that the CKF2P and CKF2C have the best results and nearly the same RMS velocity errors. The RMS velocity error of the CKF1P is slightly higher than that CKF2P and CKF2C. The RMS velocity error of the CKF1C is significantly higher than that of the other three filters. It appears that the CKF1C diverges for this maneuvering target tracking scenario. A similar pattern is observed in the results of the angular rate errors in Figure 7.

To evaluate the overall performance of a filter, we use the root time-averaged mean square (RTAMS) error [11] for position, velocity, and angular rate. Let $p_{j,i}^{t}$ and ${\hat{p}}_{j,i}^{t}$ denote the true and estimated position of the target, respectively, at time index *j* in the *i*th Monte Carlo run. The RTAMS position error [11] is defined by

(93) $${\mathrm{RTAMS}}_{\mathrm{pos}}=\sqrt{\frac{1}{N_{f}M}\sum_{j\in S_{f}}\sum_{i=1}^{M}{∥{\hat{p}}_{j,i}^{t}-p_{k,j}^{t}∥}^{2}},$$

where $S_{f}$ is a set of time indices with $N_{f}$ indices, and *M* is the number of Monte Carlo runs. We have chosen time indices from 51 to 101 to define $S_{f}$. The RTAMS error [11] for velocity and angular rate are similarly defined. Table 4 presents the RTAMS error metric for position, velocity, and angular rate for measurement error SDs of 1 mrad and 2 mrad. Results in Table 4 show that the CKF2P and CKF2C have the best RTAMS errors for position, velocity, and angular rate, which are nearly the same.

Table 5 presents CPU times for each Monte Carlo run and CPU times relative to the CKF1P. Results in Table 5 show that the CKF1P has the fastest CPU time, being slightly faster than the CKF2P.

Let $x_{k,i}$ and ${\hat{x}}_{k,i}$ be the true and filtered estimated *X*-coordinates at time k, respectively. Similar definitions apply for other position coordinates. Then, the sample position bias is given by [22,33]

(94) $$b_{\mathrm{pos},k}=\frac{1}{M}\sum_{i=1}^{M}\left[\left(x_{k,i}-{\hat{x}}_{k,i}\right)+\left(y_{k,i}-{\hat{y}}_{k,i}\right)+\left(z_{k,i}-{\hat{z}}_{k,i}\right)\right].$$

**Figure 8:**
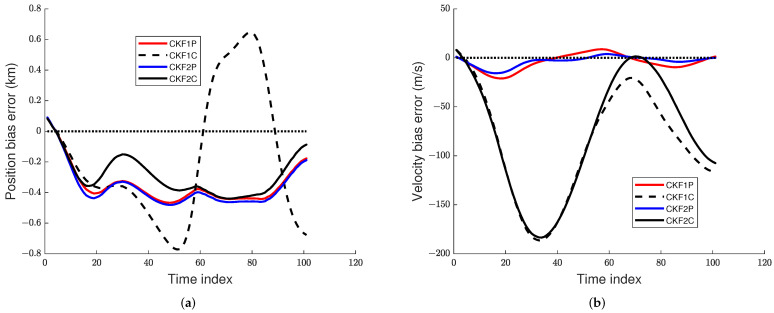
Position and velocity bias errors for the 1 mrad case. (**a**) Position bias, (**b**) Velocity bias.

**Figure 9:**
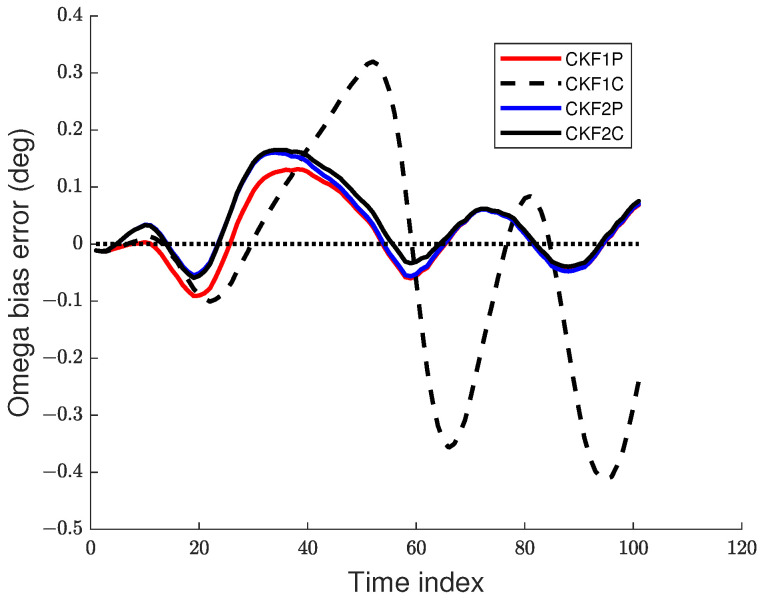
Angular rate bias error for the 1 mrad case.

For simplicity, we use “bias” to represent sample bias. Similarly, the biases for velocity and angular rate can be defined. The bias at time *k* can be positive or negative. It is desirable to have a small bias in the state estimate. The sample bias for position, velocity, and angular rate are shown in Figure 8 and Figure 9. Results in Figure 8 show that the position biases of the CKF1P, CKF2P, and CKF2C are small, and the velocity biases of CKF2P and CKF2C are nearly zero. The angular rate biases of CKF1P, CKF2P, and CKF2C become smaller with time and approach zero. The CKF1C has large position, velocity, and angular rate biases.

### 6.2. Dependence of Filtering Accuracy on the Prior Distribution

In order to analyze the dependence of filtering accuracy on the prior distribution, we have chosen a larger prior variance for the 3D polar velocity-based state relative to that used in (92)

(95) $$\begin{array}{cc} P_{0,p,2} & =\mathrm{diag}(5000^{2}\;m^{2},\;5000^{2}\;m^{2},\;90^{2}\;m^{2}s^{-2},\;\left(3*0.0873\right)\;{\mathrm{rad}}^{2},\;{\left(3*4.95\times 10^{-3}\right)}^{2}\;{\mathrm{rad}}^{2}s^{-2},\\ \; & \;500^{2}\;m^{2},15^{2}\;m^{2}s^{-2}). \end{array}$$

**Figure 10:**
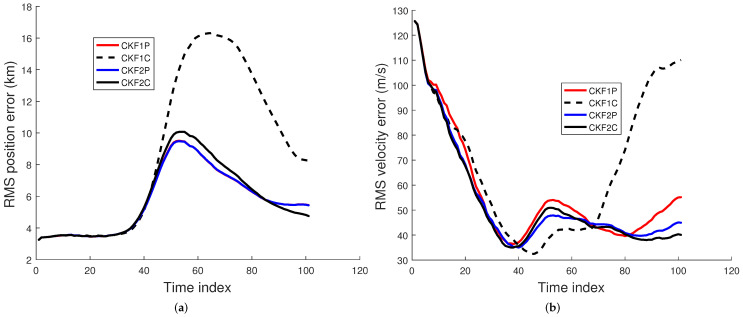
RMS position and velocity errors using the prior variance $P_{0,p,2}$ with angle SD of 1 mrad. (**a**) RMS position error, (**b**) RMS velocity error.

**Figure 11:**
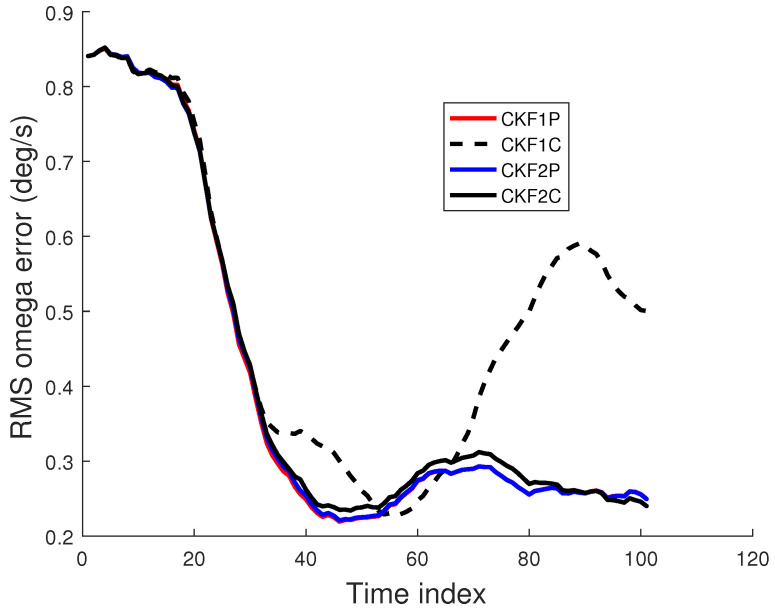
RMS angular rate error using the prior variance $P_{0,p,2}$ with angle SD of 1 mrad.

The prior variance of Cartesian position is increased by 25 times, and the other components have been increased by 9 times. The prior mean is unchanged. The RMSE plots of position, velocity, and angular rate are presented in Figure 10 and Figure 11 for the 1 mrad scenario.

Table 6 presents the RTAMS error for position, velocity, and angular rate for measurement error SDs of 1 mrad and the second prior distribution. Results in Table 6 show that the CKF2P and CKF2C have the best RTAMS errors for position, velocity, and angular rate, which are nearly the same.

### 6.3. Summary of Key Results

Based on RMS and RTAMS errors, the key results of our study are as follows:

- The CKF1P has the best position estimation accuracy. The position estimation accuracies of the CKF2P and CKF2C are close to that of the CKF1P;
- The CKF2P and CKF2C have the best velocity estimation accuracy;
- The state estimation accuracies of the CKF2P and CKF2C are comparable. However, the computational cost of the CKF2C is about twice that of the CKF2P;
- The CKF1C does not perform well for this problem and has high estimation errors.

## 7. Conclusions

We considered the challenging filtering problem of a maneuvering target in 3D using the bearing and elevation measurements from a maneuvering passive IRST sensor. Research on this problem is rather limited. The target moves with the NCT motion in the XY-plane and has an NCV motion along the *Z*-axis. We discretized the continuous-time stochastic differential equation for the NCT model using the first (Euler) and second-order Taylor approximations to obtain discrete-time NCT models. Discrete-time dynamic and measurement models are nonlinear. For each approximation, we used the polar and Cartesian velocity-based states for the NCT model. The CKF was used in each case giving rise to four filters: CKF1P, CKF1C, CKF2P, and CKF2C. Numerical results based on Monte Carlo simulations suggest that the second-order Taylor approximation-based filters CKF2P and CKF2C have the best state estimation accuracy for this scenario. Secondly, the accuracies of these two filters are nearly the same.

Our future work will develop filter initialization algorithms that can be used with real data. We shall also focus on calculating the PCRLB for the filtering problem to assess the best achievable accuracy.

## Figures and Tables

**Table 1:** Features of CKF based algorithms.

Filter	2D State in NCT	Approximation	Process Noise
CKF1P	Polar velocity	Euler	State-independent
CKF1C	Cartesian velocity	Euler	State-dependent
CKF2P	Polar velocity	TS2	State-dependent
CKF2C	Cartesian velocity	TS2	State-dependent

**Table 2:** Prior polar mean velocity-based 3D state parameters of target.

Variable	Value
${\bar{x}}_{0}$ (m)	97,580.7358
${\bar{y}}_{0}$ (m)	97,580.7358
${\bar{s}}_{0}$ (m/s)	297.0
${\bar{\theta }}_{0}$ (deg)	215.0
${\bar{\omega }}_{0}$ (deg/s)	5.672
${\bar{z}}_{0}$ (m)	9000.0
${\bar{\dot{z}}}_{0}$ (m/s)	0.0

**Table 3:** Motion profile of the sensor.

Interval (s)	Δt (s)	$\Delta \phi_{s}$ (rad)	Motion Type	Ω (rad/s)
[0,15]	15	0	CV	0
[15,31]	16	−π/4	CT	−π/64
[31,43]	12	0	CV	0
[43,75]	32	π/2	CT	π/64
[75,86]	11	0	CV	0
[86,102]	16	−π/4	CT	−π/64

**Table 4:** RTAMS position, velocity, and angular rate errors for CKF1P, CKF1C, CKF2P, and CKF2C using the prior variance $P_{0,p,1}$.

Metric	Filter	1 mrad	2 mrad
Position error (km)	CKF1P	1.137	1.355
	CKF1C	1.596	1.582
	CKF2P	1.165	1.400
	CKF2C	1.146	1.389
Velocity error (m/s)	CKF1P	28.628	28.178
	CKF1C	75.853	77.691
	CKF2P	18.959	21.132
	CKF2C	18.867	21.334
Angular rate error (deg/s)	CKF1P	0.197	0.211
	CKF1C	0.394	0.347
	CKF2P	0.197	0.214
	CKF2C	0.197	0.216

**Table 5:** CPU times (s) for each Monte Carlo run and CPU times relative to CKF1P for angle SD of 1 mrad.

Filter	CPU Time (s)	CPU Relative to CKF1P
CKF1P	0.0377	1.0000
CKF1C	0.0386	1.0129
CKF2P	0.0391	1.0356
CKF2C	0.0789	2.0910

**Table 6:** Comparison of RTAMS position, velocity, and angular rate errors for CKF1P, CKF1C, CKF2P, CKF2C using prior variances $P_{0,p,1}$ and $P_{0,p,2}$ with angle SD of 1 mrad.

Metric	Filter	$P_{0,p,1}$	$P_{0,p,2}$
Position error (km)	CKF1P	1.137	7.175
	CKF1C	1.596	13.559
	CKF2P	1.165	7.178
	CKF2C	1.146	7.454
Velocity error (m/s)	CKF1P	28.628	47.204
	CKF1C	75.853	75.192
	CKF2P	18.959	43.836
	CKF2C	18.867	42.973
Angular rate error (deg/s)	CKF1P	0.197	0.265
	CKF1C	0.394	0.440
	CKF2P	0.197	0.265
	CKF2C	0.197	0.274

## Data Availability

Not applicable.

## Abbreviations

The following abbreviations are used in this manuscript:

3D-IVKF	3D instrumental variable based Kalman filter
AOF	Angle-only filtering
ATC	Air-traffic control
BOF	Bearings-only filtering
CKF	Cubature Kalman filter
CKF1C	CKF using Euler approximation with Cartesian velocity
CKF1P	CKF using Euler approximation with polar velocity
CKF2C	CKF using order 2 weak Taylor approximation with Cartesian velocity
CKF2P	CKF using order 2 weak Taylor approximation with polar velocity
CCKF	Cartesian CKF
CEKF	Cartesian EKF
CNSKF	Cartesian new sigma point Kalman filter
CUKF	Cartesian UKF
CT	Constant turn
CV	Constant velocity
EKF	Extended Kalman filter
EKF-MSC	EKF using the MSC
EnKF	Ensemble Kalman filter
IRST	Infrared search and track
LSC	Log spherical coordinates
MPC	Modified polar coordinates
MSC	Modified spherical coordinates
MMSE	Minimum mean square error
NCT	Nearly constant turn
NCV	Nearly constant velocity
PFF	Particle flow filter
PCRLB	Posterior Cramér–Rao lower bound
PSD	Power spectral density
RP-EKF	Range-parametrized EKF
RP-UKF	Range-parametrized UKF
RMS	Root mean square
SD	Standard deviation
SDE	Stochastic differential equation
TS2	Order 2 weak Taylor
UKF	Unscented Kalman filter
UKF-MSC	UKF using the MSC

## References

[1] Aidala V.J. (1979). Kalman filter behaviour in Bearings-Only Tracking Applications. IEEE Trans. Aerosp. Electron. Syst..

[2] Allen R.R., Blackman S.S. Angle-only tracking with a MSC filter. Proceedings of the Digital Avionics Systems Conference.

[3] Asfia U., Radhakrishnan R., Sharma S.N., Gu J., Dey R., Adhikary N. (2021). Three-Dimensional Bearings-Only Target Tracking: Comparison of Few Sigma Point Kalman Filters. Communication and Control for Robotic Systems.

[4] Blackman S., Popoli R. (1999). Design and Analysis of Modern Tracking Systems.

[5] Blackman S.S., Dempster R.J., Blyth B., Durand C. Integration of Passive Ranging with Multiple Hypothesis Tracking (MHT) for Application with Angle-Only Measurements. Proceedings of the SPIE 7698, Signal and Data Processing of Small Targets 2010.

[6] Karlsson R., Gustafsson F. Range estimation using angle-only target tracking with particle filters. Proceedings of the American Control Conference.

[7] Karlsson R., Gustafsson F. (2005). Recursive Bayesian estimation: Bearings-only applications. IEE Proc.-Radar Sonar Navig..

[8] Mallick M., Morelande M., Mihaylova L., Arulampalam S., Yan Y., Mallick M., Krishnamurthy V., Vo B.-N. (2012). Angle-only Filtering in Three Dimensions. Chapter 1. Integrated Tracking, Classification, and Sensor Management: Theory and Applications.

[9] Mallick M., Chang K., Arulampalam S., Yan Y. (2019). Heterogeneous Track-to-Track Fusion in 3D Using IRST Sensor and Air MTI Radar. IEEE Trans. Aerosp. Electron. Syst..

[10] Radhakrishnan R., Bhaumik S., Tomar N.K. (2018). Gaussian sum shifted Rayleigh filter for underwater bearings-only target tracking problems. IEEE J. Ocean. Eng..

[11] Ristic B., Arulampalam S., Gordon N. (2004). Beyond the Kalman Filter.

[12] Stallard D.V. An angle-only tracking filter in modified spherical coordinates. Proceedings of the AIAA Guidance, Navigation and Control Conference.

[13] Stallard D.V. (1991). Angle-only tracking filter in modified spherical coordinates. J. Guid. Control. Dyn..

[14] Li Q., Shi L., Wang H., Guo F. (2009). Utilization of Modified Spherical Coordinates for Satellite to Satellite Bearings-Only Tracking. Chin. J. Space Sci..

[15] Maggio E., Cavallaro A. (2011). Video Tracking: Theory and Practice.

[16] Kronhamn T.R. (1998). Bearings-only Target Motion Analysis Based on a Multihypothesis Kalman Filter and Adaptive Ownship Motion Control. IEE Proc.-Radar Sonar Navig..

[17] Kronhamn T.R. Angle-only tracking of manoeuvring targets using adaptive-IMM multiple range models. Proceedings of the Radar 2002.

[18] Nardone S., Lindgren A., Gong K. (1984). Fundamental properties and performance of conventional bearings-only target motion analysis. IEEE Trans. Autom. Control.

[19] Peach N. (1995). Bearings-only Tracking using a Set of Range-parameterised Extended Kalman Filters. IEE Proc.-Control Theory Appl..

[20] Zhang Y., Lan J., Mallick M., Li X.R. (2021). Bearings-Only Filtering Using Uncorrelated Conversion Based Filters. IEEE Trans. Aerosp. Electron. Syst..

[21] Jauffret C., Pillon D. (1996). Observability in passive target motion analysis. IEEE Trans. Aerosp. Electron. Syst..

[22] Bar-Shalom Y., Li X.R., Kirubarajan T. (2001). Estimation with Applications to Tracking and Navigation.

[23] Gelb A. (1974). Applied Optimal Estimation.

[24] Hoelzer H.D., Johnson G.W., Cohen A.O. (1978). Modified Polar Coordinates—The Key to Well Behaved Bearings Only Ranging. IR&D Report 78-M19-OOOlA.

[25] Johnson G.W., Hoelzer H.D., Cohen A.O., Harrold E.F. (1979). Improved coordinates for target tracking from time delay information. J. Acoust. Soc. Am..

[26] Julier S., Uhlmann J., Durrant-Whyte H.F. (2000). A new method for the nonlinear transformation of means and covariances in filters and estimators. IEEE Trans. Autom. Control.

[27] Arasaratnam I., Haykin S. (2009). Cubature Kalman filters. IEEE Trans. Autom. Control.

[28] Alspach D., Sorenson H. (1972). Nonlinear Bayesian estimation using Gaussian sum approximations. IEEE Trans. Autom. Control.

[29] Mallick M., Mihaylova L., Arulampalam S., Yan Y. Angle-only Filtering in 3D Using Modified Spherical and Log Spherical Coordinates. Proceedings of the 14th International Conference on Information Fusion.

[30] Robinson P.N., Yin M.R. Modified spherical coordinates for radar. Proceedings of the AIAA Guidance, Navigation and Control Conference.

[31] Mallick M., Morelande M., Mihaylova L., Arulampalam S., Yan Y. Comparison of Angle-only Filtering Algorithms in 3D using Cartesian and Modified Spherical Coordinates. Proceedings of the 15th International Conference on Information Fusion.

[32] Mallick M., Morelande M., Mihaylova L. Continuous-Discrete Filtering using EKF, UKF, and PF. Proceedings of the 15th International Conference on Information Fusion.

[33] Mallick M., Arulampalam S. Comparison of Nonlinear Filtering Algorithms in Ground Moving Target Indicator (GMTI) Target Tracking. Proceedings of the SPIE 5204, Signal and Data Processing of Small Targets 2003.

[34] Mallick M., Tian X., Liu J. Evaluation of Measurement Converted KF, EKF, UKF, CKF, and PF in GMTI Filtering. Proceedings of the 10th International Conference on Control, Automation and Information Sciences (ICCAIS 2018).

[35] Radhika M.N., Mallick M., Tian X., Liu J. Performance Evaluation of IMM-based Nonlinear Filters for a Highly Maneuvering Aircraft. Proceedings of the 10th International Conference on Control, Automation and Information Sciences (ICCAIS 2021).

[36] Badriasl L., Arulampalam S., Finn A. (2018). A novel batch Bayesian WIV estimator for three-dimensional TMA using bearing and elevation measurements. IEEE Trans. Signal Process..

[37] Gupta S.D., Yu J.Y., Mallick M., Coates M., Morelande M. Comparison of Angle-only Filtering Algorithms in 3D Using EKF, UKF, PF, PFF, and Ensemble KF. Proceedings of the 18th International Conference on Information Fusion.

[38] Mallick M., Sinha A., Liu J. Enhancements to Bearing-only Filtering. Proceedings of the 20th International Conference on Information Fusion.

[39] Nguyen N.H., Doğançay K. (2018). Instrumental variable based Kalman filter algorithm for three-dimensional AOA target tracking. IEEE Signal Process. Lett..

[40] Tichavsky P., Muravchik C.H., Nehorai A. (1998). Posterior Cramér-Rao bounds for discrete-time nonlinear filtering. IEEE Trans. Signal Process..

[41] Mallick M., Arulampalam S., Yan Y. Posterior Cramér-Rao Lower Bound for Angle-only Filtering in 3D. Proceedings of the 7th International Conference on Control, Automation and Information Sciences (ICCAIS 2021).

[42] Schmitt L., Fichter W. (2019). Globally valid posterior Cramér–Rao bound for three-dimensional bearings-only filtering. IEEE Trans. Aerosp. Electron. Syst..

[43] Li X.R., Jilkov V.P. (2003). Survey of maneuvering target tracking, Part I: Dynamic Models. IEEE Trans. Aerosp. Electron. Syst..

[44] Gustafsson F., Isaksson A.J. Best choice of state variables for tracking coordinated turns. Proceedings of the 35th IEEE Conference on Decision and Control.

[45] Morelande M.R., Gordon N.J. Target tracking through a coordinated turn. Proceedings of the IEEE International Conference on Acoustics, Speech, and Signal Processing (ICASSP’05).

[46] Roth M., Hendeby G., Gustafsson F. EKF/UKF maneuvering target tracking using coordinated turn models with polar/Cartesian velocity Target tracking through a coordinated turn. Proceedings of the 17th International Conference on Information Fusion (FUSION).

[47] Li X.R., Bar-Shalom Y. (1993). Design of an interacting multiple model algorithm for air traffic control tracking. IEEE Trans. Autom. Control.

[48] Jazwinski A.H. (1970). Stochastic Processes and Filtering Theory.

[49] Särkkä S., Solin A. (2019). Applied Stochastic Differential Equations.

[50] Kloeden P.E., Platen E. (1992). Numerical Solution of Stochastic Differential Equations.

[51] Arfken G.B., Weber H.J., Harris F.E. (2013). Mathematical Methods for Physicists: A Comprehensive Guide.

[52] Horn R.A., Johnson C.R. (1991). Topics in Matrix Analysis.

[53] Yuan T., Bar-Shalom Y., Tian X. (2011). Heterogeneous Track-to-Track Fusion. J. Adv. Inf. Fusion.

[54] Arasaratnam I., Haykin S., Hurd T.R. (2010). Cubature Kalman filtering for continuous-discrete systems: Theory and simulations. IEEE Trans. Signal Process..

